# A highly cost-effective, eco-friendly tissue lysis and extraction method for faster DNA isolation from fish fin

**DOI:** 10.1371/journal.pone.0318708

**Published:** 2025-02-18

**Authors:** Pritam Lenka, Namrata Singh, Deepra Ghosh, Vivekanand Mahato, Sajalendu Ghosh

**Affiliations:** 1 Department of Zoology, Dr. Shyama Prasad Mukherjee University, Ranchi, Jharkhand, India; 2 Department of Statistics, Fox School of Business, Temple University, Philadelphia, Pennsylvania, United States of America; ICAR—Directorate of Coldwater Fisheries Research, INDIA

## Abstract

Proper DNA extraction is an essential step in molecular biology research, for various downstream applications. Several modifications have been made to the first extraction protocol by Friedrich Miescher in 1869. The current work aimed to standardize an eco-friendly and quicker DNA extraction process that could be used in resource-limited laboratories by utilizing low-priced household liquid detergents and easily accessible salt. The pectoral fin tissues were lysed at 58°C with two modified lysis buffers using detergent 1 & 2 along with the conventional lysis buffer (SDS) as control. Instead of extraction with organic solvents, a 5M edible salt solution was used. This modified protocol resulted in yielding 3269.67 (±108.7) ng/µl and 3000 (± 15) ng/µl of DNA using detergent 1 and 2 with comparable quality of DNA as confirmed by OD_260/280_, i.e., 1.7 (± 0.026) and 1.72 (± 0.015) respectively, while the conventional method gave a maximum of 2512.33 (± 45.78) ng/µl of DNA with 1.76 (± 0.021) OD_260/280_ values. The overall cost of the proposed protocol was found almost 32 times less than the conventional method. The quality of DNA obtained by the modified protocol was pure enough to be used in PCR amplification of both nuclear (microsatellite) and mitochondrial (*COX1*) DNA for further application of genotyping. This modified protocol for DNA extraction from fish fin was faster (half the time required than the SDS lysis), of comparable quality and even better quantity with significantly lesser overall cost, and can be recommended for DNA extraction from fish samples in any resource-constrained laboratories.

## Introduction

Deoxyribonucleic acid (DNA), serves as the blueprint of life, containing the genetic instructions that govern the development, function, and diversity of all living organisms, and comes under the purview of molecular genetics [1]. Most molecular genetic techniques begin with the extraction of biomolecules, especially genomic DNA [2]. The ability to extract and purify DNA from various biological sources has been a significant breakthrough in molecular biology, genetics, and biotechnology. DNA extraction methods have become the foundation for numerous scientific applications, from understanding evolutionary relationships to identifying criminals through forensic analysis. Therefore, the selection of an efficient isolation protocol is important since the downstream applications of DNA fairly depend on its quality and quantity [3].

Recent advancements in DNA extraction techniques aim to streamline the process while minimizing the environmental impact and reducing the financial burden, simultaneously allowing for higher throughput and reproducibility. The complexity of DNA extraction contributes to its time-consuming nature, as well as the higher initial cost of using highly corrosive and hazardous chemicals [4]. The process typically involves multiple steps like cell lysis, purification, and DNA recovery [5]. Each stage demands careful optimization to ensure maximum DNA yield and purity. On the other hand, newer modified methods and extraction kits require significantly less time with increased efficiency, albeit with higher initial costs. Commercial DNA extraction kits, which offer convenience and standardized protocols, can be relatively more expensive, especially when large samples need to be processed. However, the growing interest in exploring the vast genomic landscape has made these methods continuously evolve, seeking ease of purification, faster isolation timing, and overall cost reduction [6].

The Indian inland aquaculture industry is rapidly emerging, and the growing demand especially for major carp has caused the practice of congregated breeding causing unnatural hybridisation [7]. These hybrids can be better understood through advanced genetic studies, which require high-quality genomic DNA [7–9]. Several studies have reported various protocols tailored to specific objectives and tissue types [10–13]. A study back in 2017 by De Lombaert et al., suggests that fin clipping had no direct effect on the whole body as evidenced by the cortisol level [14]. According to another investigation, skin swabbing, a sophisticated technique, was found to inflict the same amount of stress as fin clipping but was not effective in terms of DNA recovery [15]. Finding a potential DNA isolation protocol for fish tissues has become a major concern which would be comparatively faster, cheaper, and eco-friendly compared to the conventional one.

The present study was so designed to develop a unique DNA extraction technique using fish fin tissue to obtain a sufficient quantity of pure DNA [14,16]. This work also explores the feasibility of replacing traditional, costly, and hazardous chemicals with more accessible, affordable, and safer alternatives for DNA extraction. This methodology intends to target research laboratories with resource-limited settings by modifying lysis buffer composition with less costly household detergents (shampoo, hand wash gel), in place of standard molecular biology grade detergents to offer potential cost savings.

## Materials and methods

### Collection and preservation of Fish tissue sample

For the proposed study the pectoral fin of the Indian major carp (Cyprinids) including *Labeo rohita*, *Labeo catla,* and *Cirrhinus mrigala* was considered, thereby following minimally invasive tissue sampling method. 2 cm^2^ of the pectoral fin was clipped off, washed in distilled water and soaked. Fin tissues were immediately suspended in absolute alcohol and stored at −20°C.

### Ethical statement

This study did not involve either human participants or higher vertebrates but rather was based on edible fish, so there is no need for clinical trial registration or ethical approval.

### Chemical alternatives

In the current study instead of using molecular biology grade detergents, less costly liquid detergents like Clinic Plus Shampoo (Detergent 1) and Dettol Handwash (Detergent 2) that are available in the common market were considered for this modified lysis buffer preparation.

Extraction was performed by using edible table salt (TATA) available in all markets instead of molecular biology grade sodium chloride (NaCl). 5M salt solution was prepared as per the molecular weight of NaCl and autoclaved.

### Preparation of lysis buffers

The necessary reagents for the preparation of lysis buffer included 50 mM Tris-HCl, 50 mM EDTA, 100 mM NaCl, and 100 µg/ml of proteinase-K, other than a molecular biology grade detergent. Two different types of lysis buffer were prepared using two different liquid detergents, simultaneously the conventional lysis buffer was prepared using 1% SDS as control.

0.5% of concentrated liquid detergents each from the Clinic plus shampoo and Dettol handwash was mixed with the necessary reagents mentioned above to prepare the type 1 and 2 lysis buffers respectively. Except for the detergents all other reagents were necessarily sterilized before preparing the lysis buffers. The concentrated liquid detergents usually contain proteases, hence considered DNase free.

### Step-wise DNA isolation protocol

#### Lysis.

600 µl of both type-1 and type-2 lysis buffers were taken separately into sterile microcentrifuge tubes (1.5 ml) and the fin tissues (2 cm^2^) were suspended in it. The tubes were mixed well and incubated at 58°C for almost 3 hours, with intermittent shaking every 30 minutes. After 3 hours, complete lysis was marked by clear lysate with separated fin rays. The tubes were then centrifuged at 16128 g for 10 minutes at room temperature and the supernatant was taken.

#### Salting-out extraction of DNA.

The lysates from both types of lysis buffer were treated with equal volumes of 5M edible salt solution and mixed gently. High salt concentration help decrease the solubility of proteins thereby separating it from the DNA [17], when centrifuged at 16128 g for 10 minutes again. The clear supernatant with dissolved DNA (around 400 µl) was collected.

#### DNA precipitation.

The supernatant was than treated with both isopropanol (0.7 vol) and ice-chilled ethonal (2 vol) separately. The tubes were than kept at −20°C for at least 30 minutes, however, could also be stored overnight at 4°C. After centrifugation at 16128 g for 10 minutes at 4°C, the supernatant was decanted and the DNA pellets were collected. Since a high concentration of salt solution was used during extraction, the DNA pellets were washed twice with 70% alcohol for proper salt mitigation. The pellets were then air dried at room temperature, followed by suspension in 80 µl TE buffer and stored immediately at −20°C for downstream processing.

### DNA extraction using the conventional method as a control

Parallelly DNA was extracted using the conventional organic extraction method to compare the efficiency of the proposed extraction method.

The fin tissue was suspended in the conventional lysis buffer containing 50 mM Tris-HCl, 50 mM EDTA, 100 mM NaCl, 1% SDS, 100 µg/ml proteinase-K and incubated at 50°C for around 6–7 hours to achieve the complete lysis. The lysate was centrifuged at 16128 g and the supernatant was transferred into a new tube. Equal volume of a mixture of phenol, chloroform, and isoamyl alcohol (P:C:I) was added to the lysate in 25:24:1 ratio and mixed well. The tube was then centrifuged at 16128 g for 10 minutes at room temperature resulting in the separation of liquid layers. The supernatant with dissolved DNA was pipetted out carefully and treated with equal volumes of 3M sodium acetate, and 2.5 volumes of pre-chilled ethanol and left at −20°C for 1 hour. The tube was again centrifuged at 16128 g and the supernatant was decanted and the pellets were washed with 70% alcohol and air dried. The dried pellets were then resuspended in 80 µl TE buffer and kept at −20°C [18–20].

### Assessment of DNA yield

To verify the quality and concentration of the DNA yield, spectrophotometric analysis and agarose gel electrophoresis were conducted. Additionally, PCR amplification was carried out to further ascertain the DNA quality.

#### Spectrophotometric analysis.

DNA dissolved solution was measured for absorbance by taking the reference solution of TE buffer as the control. The absorbance both at 260 nm and 280 nm was recorded for all DNA samples. The ratio of OD_260/280_ that decides the DNA purity was derived.

***Statistical assessment***: Summary statistics and inferential tests were carried out in the statistical software R’ Studio version Safari/605.1.15 built-in Temple University’s High-Performance Computing server. Standard t-tests were performed to establish the importance of the two new methods. The scenario was set in a multiple testing setting which urged the use of the False Discovery Rate controlling BH method [21]. *False Discovery Rate* is defined as $E\left(\frac{V}{R\vee 1}\right)$ where V is the number of hypotheses that was true but got rejected and R is the total number of hypotheses rejected; this is the most common overall measure of Type-I error rate in the literature. The *Benjamini-Hochberg (BH)* method transforms the ordered p-values $P_{\left(i\right)}$ to $\frac{d}{i}P_{\left(i\right)}$where d is the total number of hypotheses and i=1,2 and then they are compared against *α*, the desired level of significance.

#### Gel electrophoresis.

According to the DNA concentration confirmed by the Spectrophotometric analysis, the DNA bands were then visualized by 0.8% agarose gel electrophoresis. DNA samples were loaded into wells with 6X loading dye and 100 bp ladder DNA was used as a marker. DNA bands were then visualized and documented.

#### PCR amplification.

The DNA was then amplified for both nuclear microsatellite markers and mitochondrial marker gene (*COX1*) to further verify its quality. MFW1 Primer pair (**F**: 5’ GTCCAGACTGTCATCAGGAG 3’ and **R**: 5’ GAGGTGTACACTGAGTCACGC 3’) was employed to amplify the targeted microsatellite region with the following touchdown protocol [22–24]. PCR reactions were carried out in 10 µl containing 30–50 ng of template DNA, 10 µ M of each primer, 1× reaction buffer (New England Biolabs), 0.2 mM of each of dNTPs (New England Biolabs), and 0.5 U of Taq DNA polymerase (New England Biolabs) using a thermocycler (Eppendorf) with following time and temperature selections: 94°C enzyme-activating step for 5 min, followed by a touchdown program (94°C denaturing step for 30s, followed by initial annealing temperature of 70°C, subsequently run down to 54°C at 1°C decrease/cycle, 72°C extension step for 1 min), followed by a uniform three-step amplification profile (94°C denaturing step for 30s, 54°C annealing step for 30s, 72°C extension step for 1 min) for another 23 cycles, then 72°C for 10 min, and finally held at 4°C.

Similarly, the universal primer pair **F1**: 5’ TCAACCAACCACAAAGACATTGGCAC 3’ and **R1**: 5’ AGACTTCTGGGTGGCCAAAGAATCA 3’ was employed to amplify the mitochondrial *COX1* region [25]. The PCR protocol for the above amplification included an initial enzyme activation step at 95°C for 3 minutes. This was followed by an annealing step starting at 50°C, and a 72°C extension step of 45 seconds. After this, a uniform three-step amplification profile was applied for 34 additional cycles, comprising a 95°C denaturing step for 30 seconds, a 50 °C annealing step for 30 seconds, and a 72°C extension step for 3 minutes and finally held at 4°C.

## Results

### Observation of cell lysis

The lysis step was carefully observed to mark any little differences in lysis caused due to the modified the lysis buffer. The incubated tubes were checked every 30 minutes to mark the desired changes in fin tissue.

***Type-1*** lysis buffer containing the detergent-1 lysed the tissue samples within 3 hours. Complete lysis was marked by the separation of fin rays forming a clear lysate. ***Type-2*** lysis buffer containing detergent-2 also successfully lysed the tissues in 2.5 hours by the formation of clear lysate. However, this lysis buffer was seen to start lysis faster, i.e., approximately 25–30 minutes before than the type-1 lysis buffer.

Generally, lysis takes around 6–7 hours using laboratory-grade detergents at 50°C, however, this modification has successfully reduced this duration to 2.5–3 hours by regulating the incubation temperature up to 58°C.

### Observation of precipitation

Alcohol is the most common reagent that helps the DNA to get precipitated. Immediately after adding isopropanol (0.7 vol), the solution looked cloudy and the cloud disappeared by gentle inversion of the tubes followed by the formation of a thread-like DNA clump. However, the addition of ice-chilled ethanol (2 vol) did not form any cloud rather DNA clumps appeared directly. No other remarkable differences were noticed during precipitation.

### DNA quality and quantity assessment

#### Spectrophotometric analysis.

This analysis was done beforehand with gel electrophoresis to know the concentration and purity of the DNA yield by using an ultra-violet spectrum of electromagnetic radiation to pass through the DNA solution. The concentration of DNA was calculated from the OD_260_ value using the formula =  OD_260_ X dilution factor X 50. The quality of DNA obtained was also calculated by the ratio of A_260_/A_280_ values mentioned in the following table where a 1.8 value is ideal (Table 1).

**Table 1:** Result of spectrophotometric analysis of the extracted DNA.

Type	Type of extraction	A_260_	A_280_	Ratio of A_260_/A_280_	Mean ratio (SD)	Concentration. of DNA in ng/µl	Mean concentration (SD)
SDS	P: C: I	0.342	0.192	1.78	1.756 (±0.021)	2565	2512.3 (± 45.78)
		0.332	0.190	1.74		2490	
		0.331	0.189	1.75		2482	
Detergent 1	5M edible table salt	0.435	0.260	1.67	1.7 (±0.026)	3262	3269 (± 108.70)
		0.422	0.246	1.71		3165	
		0.451	0.261	1.72		3382	
Detergent 2		0.402	0.236	1.70	1.72 (±0.015)	3015	3000 (± 15)
		0.398	0.230	1.73		2985	
		0.400	0.232	1.72		3000	

SD: Standard Deviation.

***Statistical interpretation of the spectrophotometric data*:** The ratio of $\frac{{\text{A}}_{260}}{{\text{A}}_{280}}$ is 1.8 for the SDS process which is considered as a convenient standard. The two detergent-based lysis methods were tested to provide a ratio that is as good as 1.8. Assuming the three recorded values are from a $\text{N}\left(\text{μ},{\text{σ}}^{2}\right)$, we tested for each of the two detergents:


$${\text{H}}_{0}:\text{μ}=1.8\;vs\;{\text{H}}_{1}:\text{μ}\neq 1.8$$

Value of ${\text{σ}}^{2}$ is not known, hence this was estimated for each of the tests by the sample variance ${\text{s}}^{2}$. The test statistic, using $\bar{R}$. as the mean ratio was formed as


$${\text{t}}_{\text{obs}}=\frac{\surd 3\left(\bar{R}-\mu \right)}{\text{s}}$$

Under ${\text{H}}_{0}$ this follows a t-distribution with degrees of freedom 2. P-values were calculated as $2\;\times \text{ Pr }({\text{t}}_{2}>\left|{\text{t}}_{\text{obs}}\right|)$. Once the p-values were obtained, they were corrected using the Benjamini-Hochberg method so that the False Discovery Rate is controlled at α, the desired level of significance.

The following Table 2 shows the corresponding calculations:

**Table 2:** Empirical evidence presenting qualitative comparisons among the lysis methods.

Lysis buffer	Mean ratio $\left(\bar{R}\right)$	Standard deviation (s)	Test statistic $\left(t_{obs}\right)$	P-values	BH-adjusted p-values
SDS	1.756	0.02082			
Type 1	1.7	0.02645	−6.560	0.0224	0.0224
Type 2	1.7167	0.01527	−9.445	0.0110	0.0220

Choosing α to be 0.01, it was concluded that the data does not provide much evidence to suggest that the population mean is different from 1.8. Hence these two methods seem to be providing ratios as powerful as the one from SDS.

Further, the mean concentration levels spoke volumes towards the supposition that the two detergent-based methods were producing more quantity of DNA than the one based on SDS.

It was established using further tests. Allowing the random sample for SDS to be from $\text{N}\left({\text{μ}}_{1},{\text{σ}}_{1}^{2}\right)$, and the two samples from detergents 1 and 2 to be from $\text{N}\left({\text{μ}}_{2},{\text{σ}}_{2}^{2}\right)$ and $\text{N}\left({\text{μ}}_{3},{\text{σ}}_{3}^{2}\right)$ respectively, our hypotheses to be tested were:


$${\text{H}}_{01}:\mu_{1}=\mu_{2}\;vs\;{\text{H}}_{11}:\mu_{1}<\mu_{2}$$

$${\text{H}}_{02}:{\text{μ}}_{1}={\text{μ}}_{3}\;vs\;{\text{H}}_{12}:{\text{μ}}_{1}<{\text{μ}}_{3}$$

We proceeded toward t-tests using the pooled estimated variance for each of the tests:


$${\text{s}}_{1}^{2}=\frac{2\times {\text{sv}}_{1}+2\times {\text{sv}}_{2}}{4}\;and\;{\text{s}}_{2}^{2}=\frac{2\times {\text{sv}}_{1}+2\times {\text{sv}}_{3}}{4}$$

where ${\text{sv}}_{1}$, ${\text{sv}}_{2}$ and ${\text{sv}}_{3}$ are the respective sample variances from the three samples. Test statistics for the two tests are:


$${\text{t}}_{1,\text{obs}}=\frac{{\bar{X}}_{2}-{\bar{X}}_{1}}{\sqrt{\frac{2}{3}{\text{s}}_{1}^{2}}}\;and\;{\text{t}}_{2,\text{obs}}=\frac{{\bar{X}}_{3}-{\bar{X}}_{1}}{\sqrt{\frac{2}{3}{\text{s}}_{2}^{2}}}$$

where ${\bar{X}}_{1}$, ${\bar{X}}_{2}$ and ${\bar{X}}_{3}$ are the respective mean concentrations from SDS, Detergent 1, and Detergent 2. Under ${\text{H}}_{01}$ and ${\text{H}}_{02}$, the aforementioned test statistics marginally follow t-distribution with degrees of freedom 4. P-values were calculated as $\mathrm{Pr}\left({\text{t}}_{4}>{\text{t}}_{1,\text{obs}}\right)$ and $\mathrm{Pr}\left({\text{t}}_{4}>{\text{t}}_{2,\text{obs}}\right)$. The BH correction was further implemented to make sure the overall Type-I error remains under desired control (Table 3).

The following table (Table 3) shows the corresponding calculations:

**Table 3:** Empirical evidence presenting quantitative comparisons among the lysis methods.

Lysis buffer	Mean $\left(\bar{X}\right)$	Standard deviation $\left(\sqrt{sv}\right)$	Test statistic $\left(t_{\cdot ,obs}\right)$	P-values	BH-adjusted p-values
SDS	2512.33	45.78			
Type 1	3269.67	108.70	11.12097	1.859×10^−4^	1.859×10^−4^
Type 2	3000.00	15	17.53133	3.108×10^−5^	6.216×10^−5^

At our chosen level of significance α=0.01, strong evidence was found from the adjusted set of p-values that the mean concentration of DNA for both the detergent-based methods was significantly higher than the concentration from SDS.

#### Gel electrophoresis.

**Fig 1:**
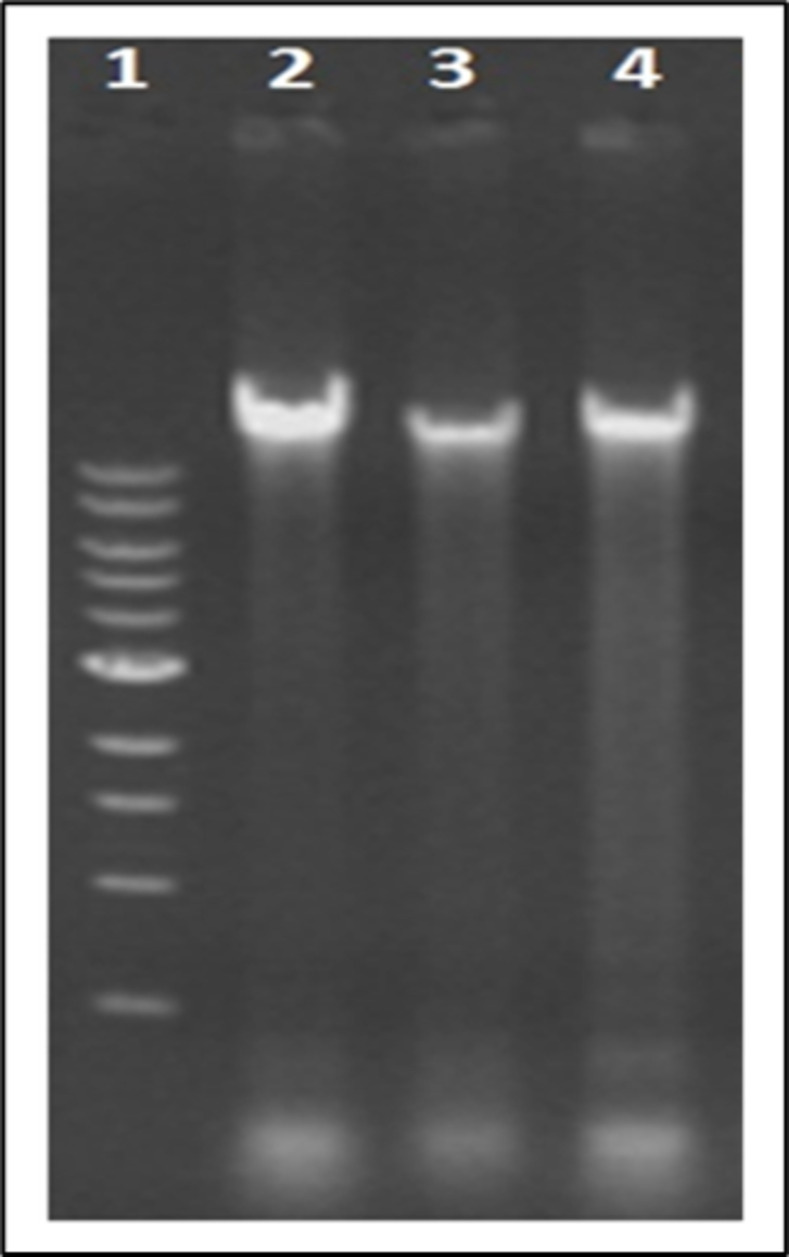
Agarose gel electrophoresis for DNA visualization. DNA bands seen in 0.8% Agarose (Lane 1 shows 100 bp DNA ladder, Lane 2 and 4 show DNA obtained by using the detergent 1 and 2 in the lysis buffer respectively, Lane- 3 show the DNA bands obtained by standard P:C:I extraction method).

The DNA bands that appeared in the agarose gel were visualized and documented under the UV trans-illuminator. The important inferences from the said electrophoretogram (Fig 1) were that detergents 1 and 2 with high concentrations of the table salt in combination were effective in isolating genomic DNA mostly free from RNA contamination comparable to the quality of DNA obtained by conventional P:C:I method. The use of RNase would certainly minimize the faint trail of RNA in the gel but can also be ignored, depending upon the downstream analysis steps.

#### PCR amplification.

**Fig 2:**
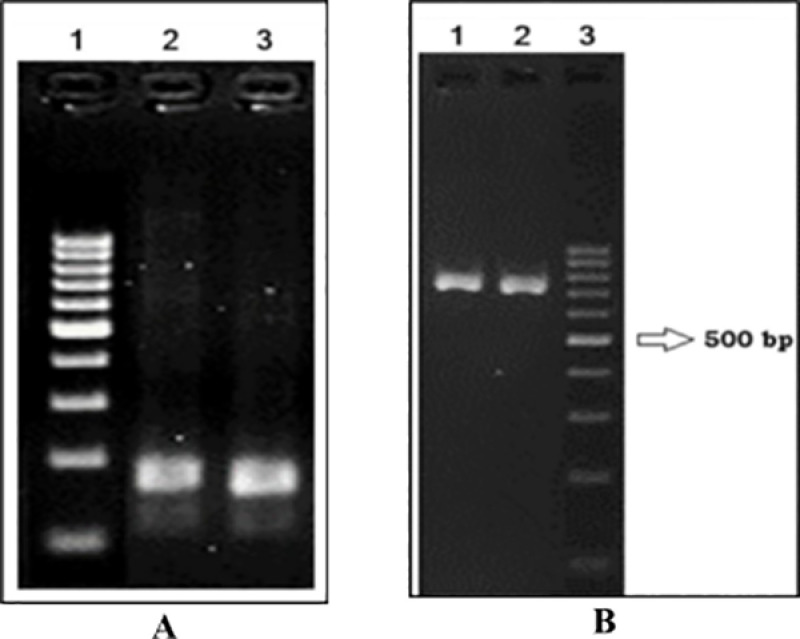
PCR amplification of both mitochondrial and nuclear genes. 2(A) The electrophoretogram showing the PCR product of the microsatellite markers (lane 1: shows the 100 bp DNA ladder, and lanes 2 and 3: show the PCR products obtained from the DNA extracted by detergent 1 and 2 respectively). 2(B) The gel shows the PCR product for *COX1* gene (lanes 1 and 2: showing successful amplification from the DNA extracted by detergent 1 and 2 respectively, lane 3: 100 bp DNA ladder).

During polymerase chain reaction (PCR), successful amplification for both nuclear microsatellite marker and mitochondrial *COX1* gene was found using the template DNA extracted by either of the two lysis buffers used in this study (Fig 2). DNA extracted using the conventional P:C:I method was not considered for PCR amplification as this was checked and reported earlier [7,11,24].

## Discussion

The work was designed initially to check whether alternative cheaper chemicals could be incorporated in place of costly and hazardous chemicals to develop an alternate extraction protocol without compromising the quality and quantity of DNA yield. Some specific changes were made to the extraction procedure to determine the objective. The modified protocol stood nowhere behind the conventional method performed parallelly as the control.

The modified lysis buffers prepared by using comparatively cheaper and safer chemical alternatives successfully lysed the fin tissues in almost half the time (2.5–3 hours) as compared to conventional lysis that typically uses SDS and takes about 6–7 hours. Regulating the incubation temperature from 50°C to 58°C was found more efficient for the modified lysis buffers for successful lysis. The previously reported protocols using laundry detergents to isolate DNA vary with different tissue types [26]. In 2005 Naisiri et al. described a protocol for isolating DNA from blood samples using laundry detergent powder [27,28]. Few studies also report the extraction of the bacterial genome using laundry detergent [29,30], while a few studies also reportedly extracted DNA from processed food products, which still holds potential challenges of fragmentation that may obstruct downstream applications [31]. This protocol seems to be the very first report using liquid detergents to isolate DNA from fish fin tissues which are generally hard and tough to digest during cell lysis.

The use of edible table salt during extraction was found to give a comparable yield to the standard salting-out extraction method [32]. The use of both isopropanol and ethanol was found to be equally effective for the precipitation of DNA. DNA cloud appeared immediately after adding isopropanol and disappeared by inverting the tubes 2–3 times gently followed by the formation of a thread-like DNA clump, however, no such cloud appeared in the case of ethanol but the DNA clump appeared directly after gentle inversion. Either of the two alternatives of precipitation can be opted as per availability for getting the same result and it is also advisable not to use any monovalent cations for precipitation since the extraction is done with high molar salt solution [33]. However, the washing step can be repeated for complete salt mitigation.

An OD_260/280_ ratio of 1.8 considered the purest form of DNA without possible contamination, is difficult to achieve [34]. The same protocol applied in repetition to extract genomic DNA from the equal size of the same tissue sample almost always ends up with different qualities of DNA showing different OD_260/280_ [35]. However, we achieved this ratio ranging from 1.67 to 1.73 which is also quite considerable and does not render the DNA unsuitable for downstream steps. The mean concentration of DNA extracted by this protocol was 3269 (± 108.70) ng/µl and 3000 (± 15) ng/µl using type 1 and 2 lysis buffers respectively (Table 1). Whereas 2512.3 (± 45.78) ng/µl of DNA with 1.756 (±0.021) OD_260/280_ was achieved by conventional SDS lysis. Execution of appropriate t-tests and modification along the pipeline of the FDR-controlling BH method revealed that DNA yield using type 1 or type 2 lysis buffers was more than that by SDS lysis, keeping the OD_260/280_ ratio in a tightly bound neighborhood of 1.8 which signifies comparable quality (Table 2). The above interpretation proves the efficacy of this modified extraction protocol by giving a comparable purity and even greater amount of genomic DNA yield than the conventional method.

The electrophoretogram has further consolidated the statement of purity for this modified protocol. After loading an equal amount of DNA into the gel, the difference seen in band intensity specifically in lanes 2 and 4 may be because of the difference in the amount of DNA extracted (Fig 1). The RNase untreated samples though showed some RNA trails, but were capable enough for successful PCR amplification which ensures its efficiency to support downstream analyses (Fig 2).

This modification also highlights the dynamic nature of laboratory methodologies, where even a slight alteration in conditions, can have intense effects on the overall process. Additionally, the protocol substitutes SDS with liquid detergents and the molecular biology grade sodium chloride (NaCl) with edible table salt as an extraction reagent which is comparatively cost-effective and safer as well. Further, the successful PCR amplifications ensured this protocol a more streamlined and economical DNA extraction process.

Ultimately the modified method is more sustainable and cost-effective and a method of choice for DNA extraction since this process reduces the overall cost of extraction by approximately 32 times than the conventional PCI method (S1 Table), which is highly relevant in the context of resource optimization. The objective of this study is not limited to resource-constrained laboratories only, however, the use of cost-effective and less hazardous reagents is always welcome in any perspective crossing the boundaries of resource limitations. The efficiency and affordability of this protocol make it worth adopting not only in research laboratories but also in educational institutions. It is suggestive to employ this method to varied samples to validate its pros and cons to a specific target tissue or organism.

## Supporting information

S1 Table Comparison of the conventional and the proposed method of DNA isolation. The comparative explanation of the conventional PCI method and the proposed modified method of DNA isolation in terms of the chemical requirement, hazardous effects, overall cost.
(DOCX)

S1 Fig Collection of tissue sample. Procurement of pectoral fin tissue samples from freshly dead fish in the market.
(DOCX)

S1 Raw Image Original uncropped gel images. Original uncropped raw images of the electrophoretogram documented right after the experimentation.
(DOCX)
