# Supplementary material for: A highly cost-effective, eco-friendly tissue lysis and extraction method for faster DNA isolation from fish fin
Source: PLoS One. 2025 Feb 18;20(2):e0318708. doi: 10.1371/journal.pone.0318708 (PMC11835239; doi:10.1371/journal.pone.0318708)
Supplement: S1 Table — The comparative explanation of the conventional PCI method and the proposed modified method of DNA isolation in terms of the chemical requirement, hazardous effects, overall cost. (DOCX) [file pone.0318708.s001.docx]

**S1 Table: Comparison of the conventional and the proposed method of DNA isolation.**

|  | **Conventional PCI method** | **Modified method** |
| --- | --- | --- |
| **Chemicals required other than common reagents** | SDS,  Molecular biology (MB) grade Salt,  RNase,  Phenol, Chloroform, Isoamyl alcohol | Clinic Plus Shampoo, Dettol hand wash,  Table salt |
| **Chemical nature whether eco-friendly or Hazardous** | SDS   - Harmful if swallowed - Toxic in contact with skin, causes skin irritation - Cause serious eye damage - May cause respiratory irritation.   Phenol   - Toxic if swallowed, in contact with skin, or if inhaled. - Causes severe skin burns and eye damage. - Suspected of causing genetic defects. - May cause damage to organs through prolonged or repeated exposure.   Chloroform   - Causes skin irritation. - Causes serious eye irritation. - May cause drowsiness or dizziness. - Suspected of causing cancer. - Causes damage to organs through prolonged or repeated exposure.   Isoamyl alcohol   - Flammable liquid and vapor. - Harmful if inhaled. - May cause skin irritation. - Repeated exposure may cause skin dryness or cracking. | The detergent and the salt used in this protocol were seen to be used as a daily basis household and are comparatively less hazardous and table salt is consumable. |
| **The overall time required for lysis** | 5-7 hours | 2.5-3 hours |
| **Overall cost except for the common reagents** | SDS: Rs. 2331 / 100 gm (HIMEDIA)  Salt (MBG): Rs. 1560 / Kg (HIMEDIA)  RNase: Rs. 2960 / 100 mg (HIMEDIA)  Phenol, Chloroform, Isoamyl alcohol: Rs. 1192 / 100 ml (HIMEDIA)  8000 Approximately | Dettol hand wash: Rs. 98 / 200 ml  Clinic Plus shampoo: Rs. 115 / 175 ml  TATA salt: Rs. 28 / 1 Kg  250 Approximately |
| **Remarks** | The conventional method was 32 times costlier than the proposed method. | The DNA extraction method of logical choice |
| *****Common reagents: Tris HCl, EDTA, Proteinase-K, Alcohol (Ethanol/ Propanol) | | |

The comparative explanation of the conventional PCI method and the proposed modified method of DNA isolation in terms of the chemical requirement, hazardous effects, overall cost.
