# Supplementary material for: A highly cost-effective, eco-friendly tissue lysis and extraction method for faster DNA isolation from fish fin
Source: PLoS One. 2025 Feb 18;20(2):e0318708. doi: 10.1371/journal.pone.0318708 (PMC11835239; doi:10.1371/journal.pone.0318708)
Supplement: S1 Fig — Procurement of pectoral fin tissue samples from freshly dead fish in the market. (DOCX) [file pone.0318708.s002.docx]

**S1 Fig: Collection of tissue sample**


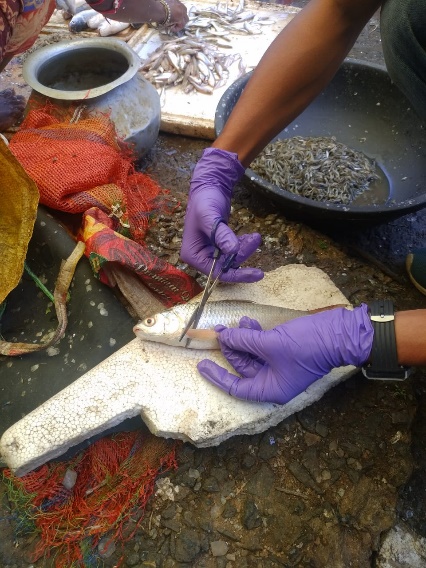


Procurement of pectoral fin tissue samples from freshly dead fish in the market.
