# Supplementary material for: A highly cost-effective, eco-friendly tissue lysis and extraction method for faster DNA isolation from fish fin
Source: PLoS One. 2025 Feb 18;20(2):e0318708. doi: 10.1371/journal.pone.0318708 (PMC11835239; doi:10.1371/journal.pone.0318708)
Supplement: S1 Raw Image — Original uncropped raw images of the electrophoretogram documented right after the experimentation. (DOCX) [file pone.0318708.s003.docx]

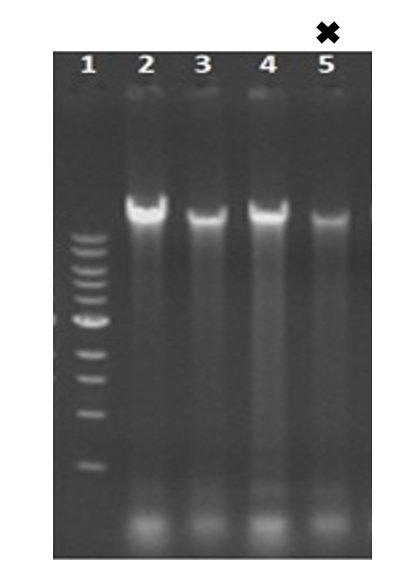


Lane 1: 100 bp DNA ladder, Lanes: 2 and 4 show DNA obtained by using the detergent 1 and 2 in the lysis buffer respectively, Lane- 3 shows the DNA bands obtained by standard SDS lysis method.


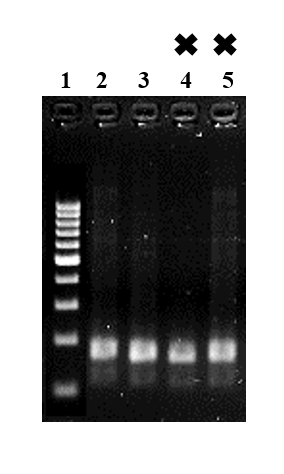


Lane 1: 100 bp DNA ladder, Lanes: 2 and 3 show PCR products (Microsatellite markers) of the DNA obtained by using detergent 1 and 2 in the lysis buffer respectively.


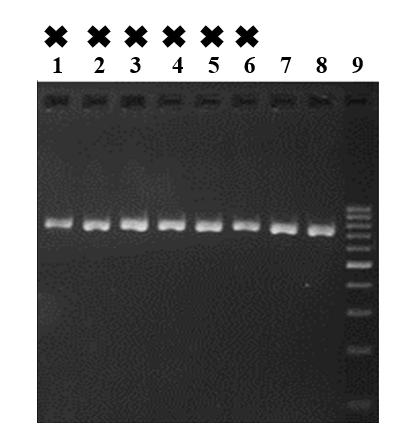


Lane 9: 100 bp DNA ladder, Lanes: 7 and 8 show PCR products (*COX1*) of the DNA obtained by using detergent 1 and 2 in the lysis buffer respectively.

**100 bp DNA Ladder**
